# Supplementary material for: First-Principles Study of Adsorption of CH4 on a Fluorinated Model NiF2 Surface
Source: Materials (Basel). 2024 Apr 27;17(9):2062. doi: 10.3390/ma17092062 (PMC11084772; doi:10.3390/ma17092062)
Supplement: Supplementary file 1 [file materials-17-02062-s001.zip › materials-2960233-supplementary.pdf]

Supporting Information

First-Principles Study of Adsorption of CH_4 on a Fluorinated Model NiF_2 Surface

T. Lindič and B. Paulus

List of Figures

Figure S1 Adsorbed structures for the adsorption of CH_4. 5
Figure S2 Adsorbed structures belonging to group I. 11
Figure S3 Adsorbed structures belonging to group II. 17
Figure S4 Adsorbed structures belonging to group III. 20
Figure S5 Adsorbed structures belonging to group IV. 22
Figure S6 Surface energies of different cuts of NiF_2 surface versus the potential calculated via the computational hydrogen electrode. The surface energies of the Ni^{2+} to Ni^{3+} transition are taken from reference [22]. The same methodology as in this reference was applied to calculate the surface energy of $NiF_2(F_2)$ (001) surface, shown with dashed line. . . 26
Figure S7 Imaginary vibrational mode displacements of the calculated transition state (image 4 in Figure 7 in the manuscript), with movement of atoms depicted by red arrows. . . . 27

List of Tables

Table S1 Adsorption energies and structural parameters for the adsorption of CH_4. N.B.: d(NiF/HF) may represent the Ni−F or H−F distance and should be interpreted together with the figure of the appropriate structure; the same holds true for d(CF/HF). 4
Table S2 Magnetic moments on the surface nickel, fluorine and the adsorbate. 6
Table S3 Charge transfer on the surface nickel, fluorine and the adsorbate for adsorbed CH_4. . . 6
Table S4 Calculated frequencies for the adsorption of CH_4 6
Table S5 Adsorption energies and bond distances for the adsorbed structures in group I. 7
Table S6 Magnetic moments on the surface nickel, fluorine and on the adsorbates for group I. . 12
Table S7 Charge transfer for the surface nickel, fluorine and the adsorbates for group I. 12
Table S8 Calculated frequencies for group I. 13
Table S9 Adsorption energies and bond distances for the adsorbed structures in group II. 17
Table S10 Magnetic moments on the surface nickel, fluorine and on the adsorbates for group II. . 17
Table S11 Charge transfer for the surface nickel, fluorine and the adsorbates for group II. 17
Table S12 Calculated frequencies for group II 17
Table S13 Adsorption energies and bond distances for the adsorbed structures in group III. . . . 18
Table S14 Magnetic moments on the surface nickel, fluorine and on the adsorbates for group III. 18
Table S15 Charge transfer for the surface nickel, fluorine and the adsorbates for group III. 18

Table S16 Calculated frequencies for group III 19
Table S17 Adsorption energies and bond distances for the adsorbed structures in group IV. 20
Table S18 Magnetic moments on the surface nickel, fluorine and on the adsorbates for group IV. 23
Table S19 Charge transfer for the surface nickel, fluorine and the adsorbates for group IV. 23
Table S20 Calculated frequencies for group IV 24

On the following pages numerical results belonging to the paper "First-Principles Study of Adsorption of CH_4 on a Fluorinated Model NiF_2 Surface" are collected. For the adsorption of CH_4 the methane molecule was placed on the surface at six different higher symmetry positions and three different distances (1.5, 1.7 and 2.0 Å, respectively) which is indicated in the tables. For the study of co-adsorption of CH_4 and HF only one distance from the surface was considered. All possible combinations of methane and HF on the 6 higher symmetry positions were considered. Furthermore, three different orientations of the HF molecule were considered, namely flat, F down and F up (with respect to the surface). The starting orientations are indicated in the tables.

All the presented results are the calculations done in VASP within the framework of spin unrestricted PBE+U D3(BJ) approach, with the kinetic cut-off energy of 700 eV and the K-point mesh of 8×8×1. More detailed model details and computational settings are described in the main text.

The following data are presented in the tables:

- #: numbering of the structure in each group
- Distance: distance of the adsorbates from the surface in the starting structure
- Position: position of the adsorbates on the surface; in case of co-adsorption the first number indicates position of CH_4 and the second the position of HF
- Orientation: u - up, d - down, f - flat; regarding the orientation of co-adsorbed HF as explained in the main text
- E_{ads}: adsorption energy as defined in the main text, in eV
- $d(X_1X_2)$: bond length between element X_1 and X_2, where X is the element symbol; in Å
- magnetic moments are in the units of μ_B
- charge transfer, as defined in the main text, in the units of e
- vibrational frequencies are in the units of cm^{-1}

A careful reader might notice the presence of some low imaginary frequencies for some of the structures. These mostly occur for weakly physisorbed cases where many possible rotations of adsorbates with low energy barriers are possible. It is important to note that these specific structures do not represent the energetically most favourable configurations. Consequently, the substantial computational cost required to refine these structures and eliminate the imaginary frequencies was considered disproportionate to the potential gain in scientific understanding and thus, deemed not justifiable.

1 Adsorption of CH_4

Table S1: Adsorption energies and structural parameters for the adsorption of CH_4. N.B.: d(NiF/HF) may represent the Ni−F or H−F distance and should be interpreted together with the figure of the appropriate structure; the same holds true for d(CF/HF).

#	Distance	Position	E_{ads}	d(NiF)	d(NiF/HF)	d(CH)	d(CH)	d(CH)	d(CF/HF)
1	1.7	0	-10.2826	2.067	2.156	1.091	1.094	1.096	1.433
2	1.7	3	-10.2822	2.067	2.156	1.092	1.093	1.096	1.433
3	1.7	5	-10.2518	2.054	2.206	1.092	1.093	1.096	1.436
4	1.7	2	-9.2965	2.275	0.984	1.088	1.089	1.093	1.456
5	1.5	5	-8.5881	1.828	1.030	1.094	1.094	1.095	1.467
6	2.0	5	-8.5864	1.833	1.027	1.094	1.095	1.096	1.468
7	2.0	1	-8.5859	1.826	1.030	1.094	1.094	1.095	1.467
8	1.7	4	-8.5826	1.835	1.025	1.093	1.095	1.096	1.468
9	1.7	1	-8.4467	1.825	1.026	1.094	1.094	1.097	1.457
10	2.0	2	-8.4459	1.826	1.025	1.094	1.094	1.097	1.456
11	1.5	3	-0.2111	1.879	1.880	1.096	1.096	1.096	1.097
12	2.0	3	-0.2035	1.876	1.883	1.095	1.096	1.096	1.097
13	1.5	4	-0.1765	1.877	1.883	1.095	1.096	1.096	1.097
14	2.0	0	-0.1736	1.872	1.889	1.096	1.096	1.096	1.097
15	1.5	0	-0.1722	1.867	1.893	1.096	1.096	1.096	1.097
16	2.0	4	-0.1703	1.878	1.882	1.096	1.096	1.097	1.097

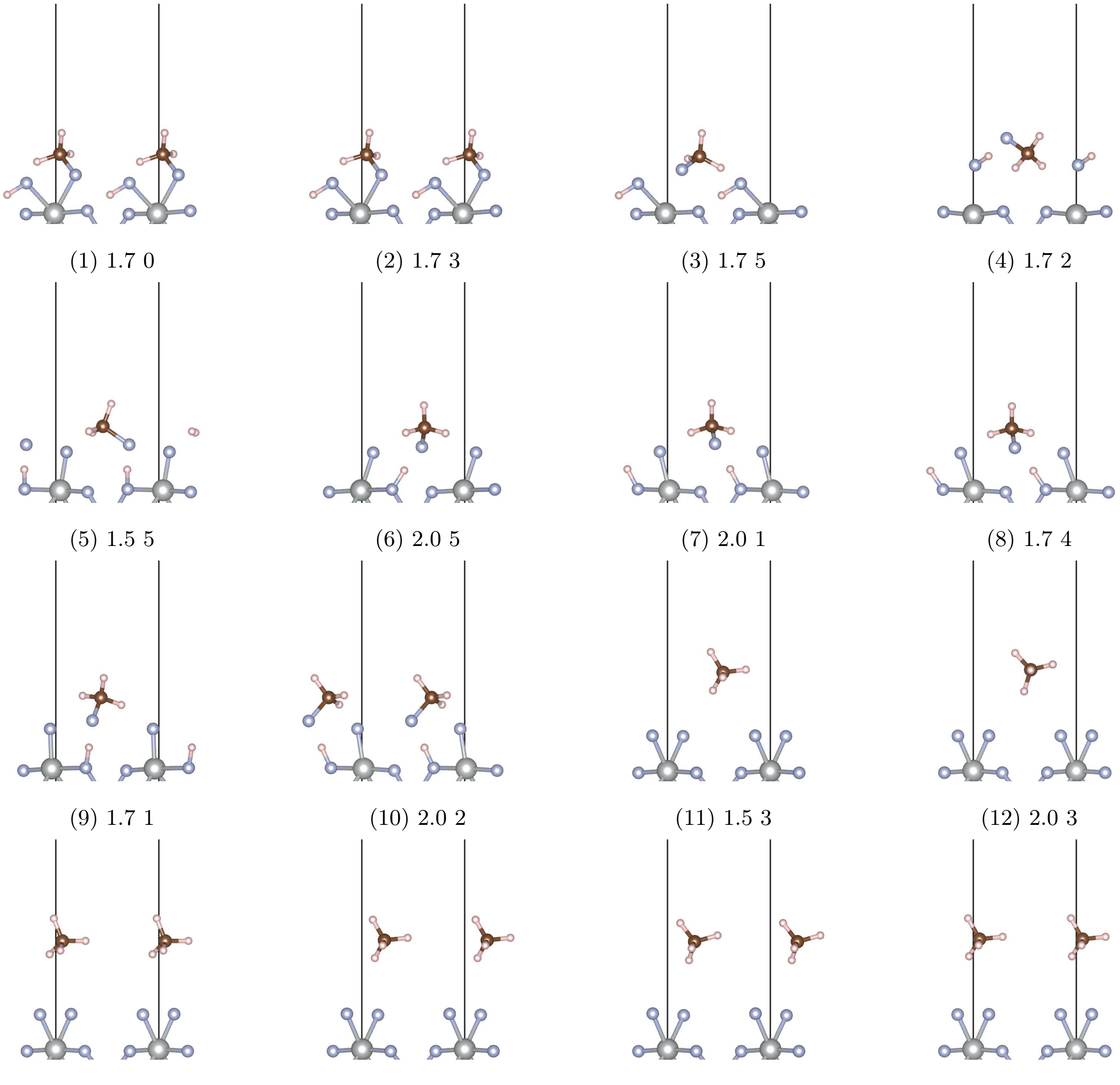

(1) 1.7 0 (2) 1.7 3 (3) 1.7 5 (4) 1.7 2

(5) 1.5 5 (6) 2.0 5 (7) 2.0 1 (8) 1.7 4

(9) 1.7 1 (10) 2.0 2 (11) 1.5 3 (12) 2.0 3

(13) 1.5 4 (14) 2.0 0 (15) 1.5 0 (16) 2.0 4

Figure S1: Adsorbed structures for the adsorption of CH_4.

Table S2: Magnetic moments on the surface nickel, fluorine and the adsorbate.

#	Distance	Position	Ni	F	F	C	H	H	H	H
1	1.7	0	-1.827	-0.014	-0.022	-0.0	-0.0	-0.0	-0.0	-0.0
2	1.7	3	-1.827	-0.014	-0.022	-0.0	-0.0	-0.0	-0.0	-0.0
3	1.7	5	-1.828	-0.01	-0.023	-0.001	-0.0	-0.0	0.0	-0.0
4	1.7	2	-1.819	-0.008	-0.0	-0.0	-0.0	-0.0	-0.0	-0.0
5	1.5	5	-1.818	-0.06	-0.0	-0.0	-0.0	0.0	0.0	0.0
6	2.0	5	-1.819	-0.057	-0.0	-0.0	-0.0	0.0	0.0	0.0
7	2.0	1	-1.818	-0.0	-0.06	-0.0	0.0	-0.0	0.0	0.0
8	1.7	4	-1.819	-0.001	-0.056	-0.0	0.0	-0.0	0.0	0.0
9	1.7	1	-1.819	0.0	-0.062	-0.0	-0.0	-0.0	-0.0	0.0
10	2.0	2	-1.819	0.0	-0.062	-0.0	-0.0	-0.0	-0.0	0.0
11	1.5	3	-2.218	-0.582	-0.59	-0.002	-0.002	-0.001	-0.001	-0.001
12	2.0	3	-2.218	-0.585	-0.588	-0.002	-0.001	-0.001	-0.001	-0.001
13	1.5	4	-2.219	-0.587	-0.587	-0.0	-0.0	-0.0	-0.0	-0.0
14	2.0	0	-2.218	-0.585	-0.589	-0.001	-0.0	-0.0	-0.0	-0.001
15	1.5	0	-2.218	-0.582	-0.59	-0.001	-0.0	-0.0	-0.0	-0.001
16	2.0	4	-2.219	-0.587	-0.586	-0.0	-0.0	-0.0	-0.0	-0.0

Table S3: Charge transfer on the surface nickel, fluorine and the adsorbate for adsorbed CH_4.

#	Distance	Position	Ni	F	F	C	H	H	H	H
1	1.7	0	0.279	0.349	0.494	-0.534	-0.009	-0.044	-0.081	-0.708
2	1.7	3	0.285	0.346	0.493	-0.487	-0.032	-0.052	-0.095	-0.706
3	1.7	5	0.281	0.362	0.493	-0.475	-0.033	-0.105	-0.063	-0.712
4	1.7	2	0.294	0.491	0.378	-0.496	-0.077	-0.066	-0.044	-0.727
5	1.5	5	0.314	0.449	0.371	-0.370	-0.142	-0.017	-0.151	-0.725
6	2.0	5	0.314	0.450	0.368	-0.411	-0.118	-0.122	-0.025	-0.735
7	2.0	1	0.314	0.349	0.467	-0.393	-0.036	-0.125	-0.123	-0.732
8	1.7	4	0.311	0.349	0.470	-0.424	-0.009	-0.117	-0.126	-0.730
9	1.7	1	0.315	0.340	0.467	-0.420	-0.020	-0.158	-0.072	-0.730
10	2.0	2	0.316	0.340	0.467	-0.431	-0.087	-0.126	-0.027	-0.731
11	1.5	3	0.000	0.010	0.014	-0.040	-0.008	-0.008	0.025	0.006
12	2.0	3	0.000	-0.004	0.026	-0.068	0.029	0.013	0.009	-0.005
13	1.5	4	-0.002	0.002	0.019	-0.032	0.011	-0.010	0.002	0.011
14	2.0	0	-0.002	0.009	0.013	-0.013	-0.007	-0.003	0.007	-0.004
15	1.5	0	0.003	0.006	0.015	-0.018	-0.013	0.014	-0.006	0.001
16	2.0	4	-0.001	0.007	0.013	-0.044	0.007	0.013	0.017	-0.011

Table S4: Calculated frequencies for the adsorption of CH_4 .

0	3	5	2	5	5	1	4
1.7	1.7	1.7	1.7	1.5	2.0	2.0	1.7
3142.13	3140.05	3141.49	3181.78	3121.76	3120.42	3119.13	3121.7
3106.18	3107.07	3109.12	3159.0	3098.35	3094.3	3098.06	3095.03
3009.25	3009.0	3011.44	3074.77	2994.24	2990.57	2993.23	2991.35
2133.2	2137.64	2212.01	3042.55	2416.4	2435.79	2411.11	2452.02
1434.77	1434.09	1457.19	1428.68	1474.6	1471.88	1472.73	1472.82
1425.44	1423.3	1429.97	1427.59	1438.15	1438.58	1437.37	1438.15
1419.14	1418.48	1418.39	1392.71	1428.85	1428.11	1429.39	1427.63
1206.73	1205.92	1194.89	1139.79	1173.44	1171.91	1172.45	1171.17
1157.15	1156.71	1152.52	1117.09	1150.47	1150.86	1149.16	1150.22
1145.88	1144.96	1148.25	943.8	1036.59	1032.92	1040.08	1028.49
1028.76	1027.23	1027.75	852.37	975.35	972.75	972.38	969.39

Continued on next page

938.59	939.07	923.67	825.15	850.26	847.92	849.51	845.23
369.83	369.29	369.19	444.79	472.86	467.7	477.04	467.08
252.48	253.12	248.99	277.4	285.55	286.34	282.71	278.68
203.17	202.52	200.43	148.87	201.53	203.48	199.0	203.19
180.15	179.67	171.01	140.8	180.89	185.59	181.73	189.0
143.23	141.78	163.55	98.19	160.77	151.99	158.01	150.42
111.58	108.98	129.29	62.01	119.82	121.52	118.59	125.93
97.28	95.9	111.39	20.15	96.51	96.93	92.64	92.01
84.84	73.77	82.56	27.5i	75.67	87.97	75.73	84.59
57.15	43.77	62.7	107.07i	31.09	29.84	34.29	35.04i
1	2	3	3	4	0	0	4
1.7	2.0	1.5	2.0	1.5	2.0	1.5	2.0
3124.23	3120.67	3071.65	3057.0	3079.85	3074.63	3070.38	3073.4
3097.68	3094.84	3046.05	3047.09	3047.02	3046.45	3042.9	3050.06
2990.78	2988.06	2998.96	2993.96	3002.9	3002.1	2996.92	2989.45
2461.51	2466.79	2963.79	2963.43	2966.3	2966.85	2965.24	2965.74
1469.44	1468.7	1351.47	1327.9	1391.09	1390.78	1399.52	1395.48
1433.29	1432.37	1298.71	1281.8	1297.42	1304.12	1277.27	1280.47
1403.67	1403.81	1232.45	1218.08	1248.62	1243.79	1243.2	1236.94
1177.69	1178.25	1185.74	1188.4	1193.43	1197.24	1197.08	1187.52
1153.22	1153.54	1094.76	1099.13	1080.55	1093.4	1096.12	1061.13
1076.58	1075.29	469.6	469.78	469.15	467.96	468.49	468.66
916.44	910.44	244.75	244.64	243.67	250.06	256.29	242.16
882.56	886.79	230.16	231.72	235.24	232.21	233.02	234.43
472.49	470.72	137.5	140.02	139.38	143.52	144.77	141.71
279.75	275.88	131.75	136.05	137.05	142.1	136.56	136.71
183.42	186.57	70.32	73.61	81.46	77.45	83.03	70.09
162.68	158.87	64.96	65.72	72.19	66.33	62.7	65.71
149.81	143.26	61.71	33.24	53.57	28.89	19.81	23.71
126.39	129.04	34.98	8.17i	8.78	15.94	8.14i	32.26i
82.9	78.84	13.07	48.41i	25.85i	25.2i	42.96i	43.22i
64.53	57.59	23.83i	56.84i	49.1i	44.02i	53.88i	66.65i
32.86	41.97	60.72i	83.49i	66.38i	63.78i	71.89i	76.49i

2 Co-adsorption of CH_4 and HF

2.1 Group I

Table S5: Adsorption energies and bond distances for the adsorbed structures in group I.

#	Orientation	Position	E_{ads}	d(CH)	d(CH)	d(CH)	d(CF)	d(NiF)	d(NiF)	d(HF)	d(HF)
1	d	5_1	-11.5292	1.096	1.097	1.097	1.413	2.030	2.033	1.013	1.013
2	u	2_5	-11.5231	1.097	1.097	1.098	1.416	2.034	2.038	1.011	1.011
3	f	4_1	-11.4700	1.097	1.097	1.097	1.414	2.034	2.038	1.010	1.011
4	u	0_4	-11.4406	1.097	1.097	1.097	1.415	2.033	2.039	1.010	1.012

Continued on next page

#	Orientation	Position	E_{ads}	d(CH)	d(CH)	d(CH)	d(CF)	d(NiF)	d(NiF)	d(HF)	d(HF)
5	d	4_0	-11.2778	1.092	1.092	1.093	1.452	2.068	2.110	1.118	0.965
6	u	4_2	-11.2673	1.091	1.093	1.093	1.453	2.089	2.106	1.104	0.961
7	f	4_2	-11.2670	1.091	1.093	1.093	1.453	2.088	2.107	1.104	0.961
8	u	4_0	-11.2132	1.097	1.098	1.098	1.414	2.040	2.044	0.991	0.988
9	u	0_5	-11.1536	1.092	1.093	1.094	1.452	2.212	2.246	1.088	0.961
10	f	3_5	-11.1535	1.092	1.093	1.094	1.452	2.215	2.244	1.087	0.961
11	d	4_1	-11.1425	1.092	1.093	1.094	1.442	2.082	2.105	1.063	0.968
12	f	0_1	-11.1355	1.092	1.092	1.093	1.449	2.203	2.249	1.090	0.963
13	f	4_5	-11.1253	1.093	1.094	1.095	1.441	2.086	2.106	1.058	0.968
14	f	1_3	-11.1235	1.092	1.093	1.095	1.441	2.087	2.107	1.060	0.968
15	f	4_3	-11.1234	1.092	1.093	1.095	1.441	2.088	2.106	1.059	0.968
16	u	0_1	-11.1219	1.092	1.093	1.095	1.441	2.088	2.104	1.060	0.968
17	u	4_3	-11.1167	1.092	1.093	1.094	1.442	2.089	2.102	1.060	0.968
18	u	5_1	-11.0900	1.091	1.093	1.095	1.440	2.061	2.146	1.057	0.966
19	u	2_1	-11.0536	1.096	1.097	1.098	1.416	2.079	2.119	1.067	0.967
20	f	4_0	-11.0093	1.096	1.097	1.098	1.417	2.085	2.122	1.065	0.967
21	f	2_4	-11.0076	1.093	1.095	1.096	1.436	2.072	2.126	1.047	0.962
22	d	0_1	-10.9160	1.097	1.097	1.097	1.416	2.219	2.220	1.088	0.978
23	f	3_1	-10.8636	1.091	1.094	1.095	1.441	2.150	2.349	1.048	0.963
24	f	1_0	-10.8488	1.093	1.094	1.094	1.442	2.200	2.303	1.052	0.964
25	u	1_5	-10.8109	1.092	1.096	1.096	1.430	2.164	2.304	1.040	0.960
26	u	4_1	-10.2132	1.092	1.095	1.095	1.443	2.079	2.156	1.055	0.965
27	f	0_5	-10.1197	1.097	1.097	1.097	1.415	2.034	2.045	1.011	1.012
28	d	0_4	-10.0996	1.089	1.090	1.092	1.490	1.873	2.455	1.011	0.996
29	u	3_4	-10.0384	1.089	1.090	1.092	1.490	1.874	2.495	0.996	1.017
30	u	3_2	-10.0369	1.089	1.089	1.091	1.490	1.876	2.466	0.997	1.015
31	f	0_3	-10.0356	1.088	1.093	1.093	1.472	2.282	-	0.990	0.990
32	f	5_1	-10.0353	1.087	1.093	1.093	1.472	2.280	-	0.990	0.990
33	d	5_4	-10.0081	1.090	1.091	1.092	1.481	1.860	-	0.990	1.043
34	d	5_0	-10.0074	1.091	1.091	1.092	1.481	1.861	-	0.990	1.044
35	d	2_0	-10.0041	1.089	1.091	1.092	1.489	1.863	-	0.987	1.035
36	d	2_4	-10.0040	1.090	1.091	1.092	1.480	1.859	-	0.988	1.047
37	u	3_0	-9.9971	1.087	1.093	1.093	1.475	2.161	-	0.993	1.000
38	u	5_0	-9.9965	1.087	1.093	1.093	1.475	2.158	-	0.992	1.000
39	u	5_3	-9.9962	1.088	1.093	1.093	1.475	2.166	-	0.993	1.000
40	u	2_0	-9.9467	1.089	1.091	1.091	1.488	1.864	-	0.986	1.043
41	u	2_4	-9.9467	1.089	1.091	1.091	1.488	1.864	-	0.986	1.043
42	f	0_4	-9.9464	1.089	1.091	1.091	1.491	1.868	-	0.988	1.037
43	u	1_0	-9.9128	1.086	1.087	1.094	1.475	2.139	-	0.996	1.000
44	f	3_0	-9.9115	1.089	1.091	1.091	1.491	1.868	-	0.988	1.038
45	u	3_1	-9.8759	1.089	1.089	1.090	1.491	1.868	-	0.981	1.041
46	f	2_1	-9.6577	1.091	1.093	1.094	1.478	2.287	-	0.949	0.973
47	f	2_5	-9.4962	1.097	1.097	1.097	1.417	2.221	-	0.974	1.073
48	f	2_0	-9.2559	1.089	1.090	1.092	1.488	1.864	-	0.987	1.042
49	f	3_4	-9.0016	1.091	1.092	1.093	1.449	2.189	-	1.087	0.960
50	u	2_3	-8.6131	1.087	1.093	1.093	1.475	2.159	-	0.993	1.000

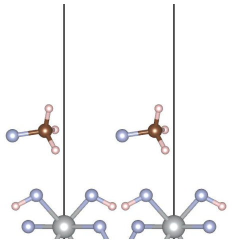

(1) d 5_1

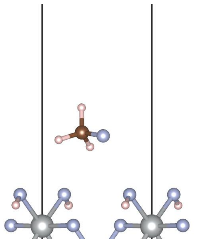

(2) u 2_5

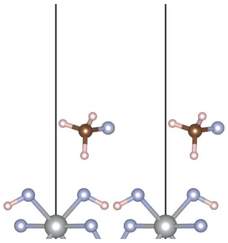

(3) f 4_1

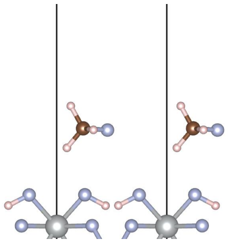

(4) u 0_4

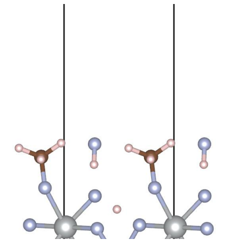

(5) d 4_0

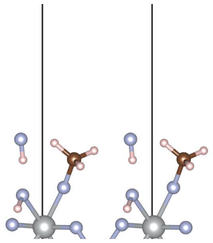

(6) u 4_2

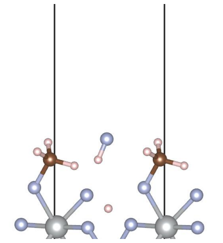

(7) f 4_2

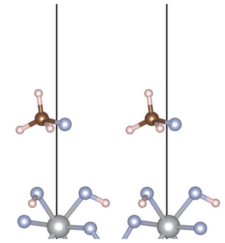

(8) u 4_0

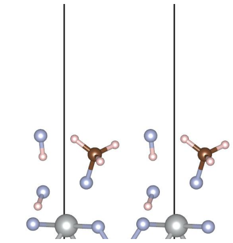

(9) u 0_5

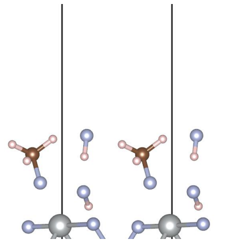

(10) f 3_5

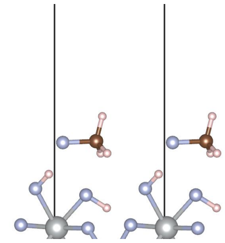

(11) d 4_1

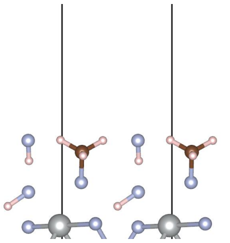

(12) f 0_1

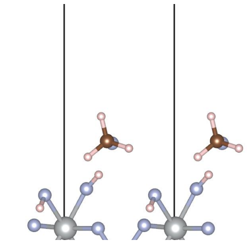

(13) f 4_5

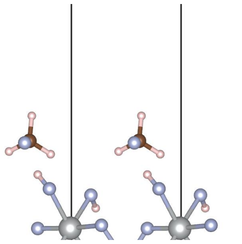

(14) f 1_3

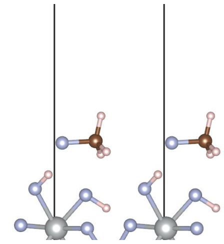

(15) f 4_3

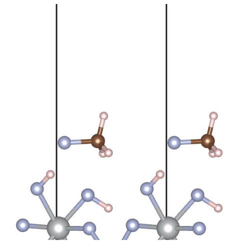

(16) u 0_1

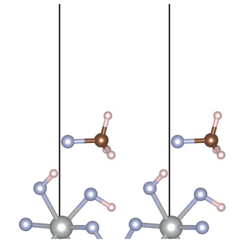

(17) u 4_3

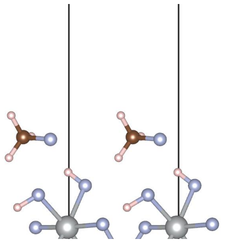

(18) u 5_1

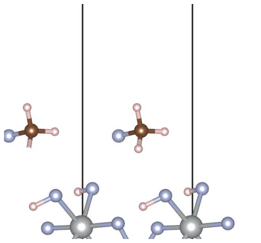

(19) u 2_1

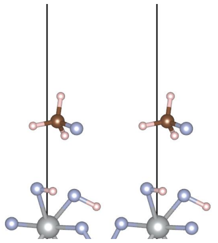

(20) f 4_0

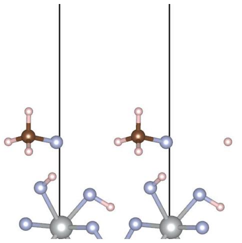

(21) f 2_4

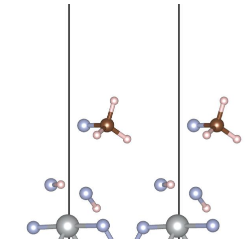

(22) d 0_1

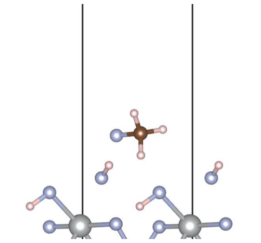

(23) f 3_1

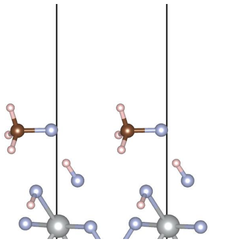

(24) f 1_0

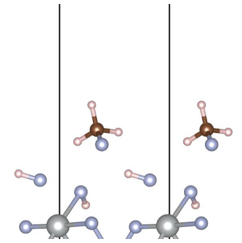

(25) u 1_5

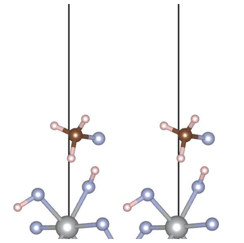

(26) u 4_1

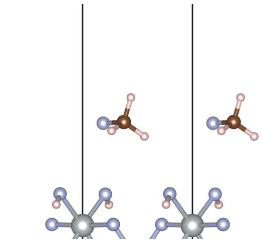

(27) f 0_5

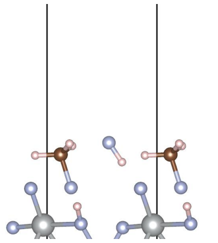

(28) d 0_4

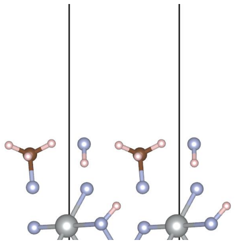

(29) u 3_4

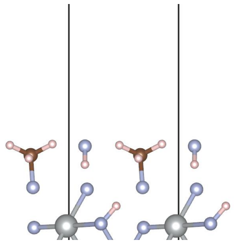

(30) u 3_2

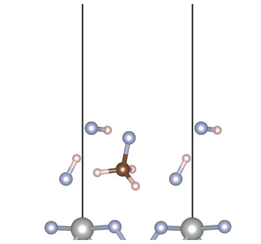

(31) f 0_3

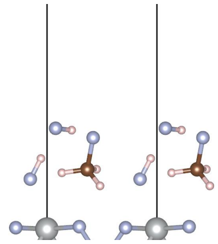

(32) f 5_1

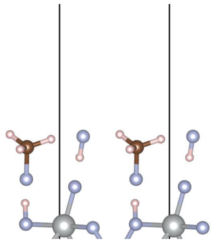

(33) d 5_4

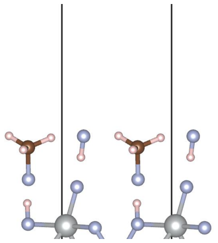

(34) d 5_0

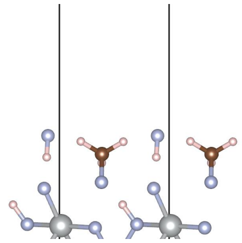

(35) d 2_0

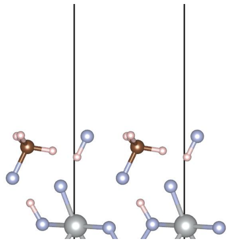

(36) d 2_4

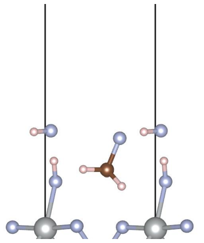

(37) u 3_0

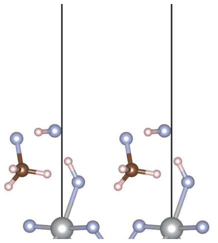

(38) u 5_0

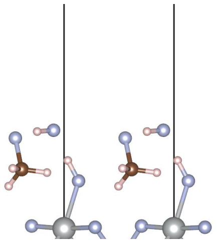

(39) u 5_3

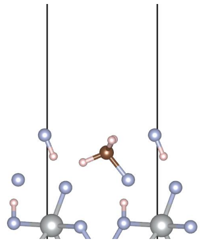

(40) u 2_0

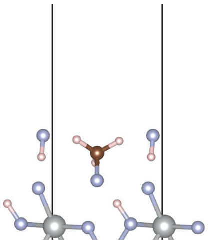

(41) u 2_4

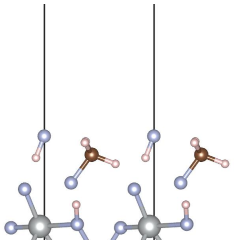

(42) f 0_4

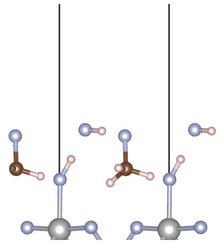

(43) u 1_0

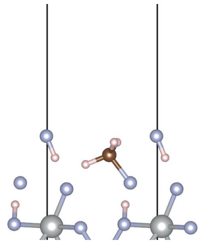

(44) f 3_0

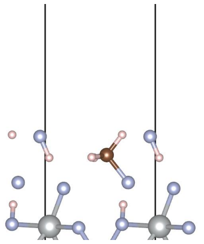

(45) u 3_1

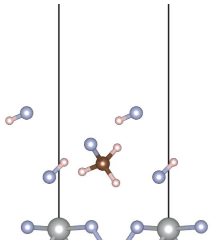

(46) f 2_1

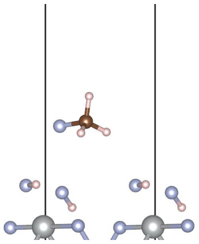

(47) f 2_5

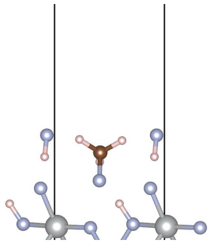

(48) f 2_0

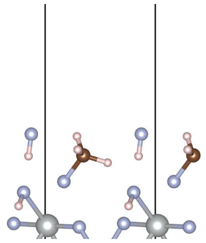

(49) f 3_4

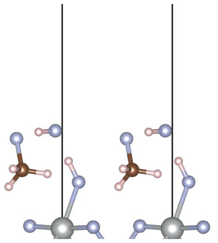

(50) u 2_3

Figure S2: Adsorbed structures belonging to group I.

Table S6: Magnetic moments on the surface nickel, fluorine and on the adsorbates for group I.

#	Orientation	Position	Ni	F	F	C	H	H	H	H	H	F
1	d	5_1	-1.837	-0.023	-0.022	-0.000	-0.000	-0.000	-0.000	-0.000	-0.000	-0.000
2	u	2_5	-1.837	-0.022	-0.022	-0.000	-0.000	-0.000	0.000	-0.000	-0.000	-0.000
3	f	4_1	-1.837	-0.022	-0.022	-0.000	-0.000	0.000	-0.000	-0.000	-0.000	-0.000
4	u	0_4	-1.837	-0.022	-0.022	-0.000	-0.000	-0.000	-0.000	-0.000	-0.000	-0.000
5	d	4_0	-1.830	-0.020	-0.014	-0.001	-0.000	-0.000	-0.001	-0.000	-0.000	-0.001
6	u	4_2	-1.830	-0.016	-0.019	-0.001	-0.000	-0.000	-0.000	-0.000	-0.001	-0.000
7	f	4_2	-1.830	-0.019	-0.016	-0.001	-0.000	-0.000	-0.000	-0.000	-0.000	-0.001
8	u	4_0	-1.831	-0.022	-0.022	-0.000	-0.000	-0.000	-0.000	-0.000	-0.000	-0.000
9	u	0_5	-1.833	-0.013	-0.016	-0.000	-0.000	-0.000	-0.000	-0.000	-0.001	-0.000
10	f	3_5	-1.833	-0.016	-0.013	-0.000	-0.000	-0.000	-0.000	-0.000	-0.000	-0.001
11	d	4_1	-1.830	-0.021	-0.014	-0.000	-0.000	-0.000	-0.000	-0.000	-0.000	-0.000
12	f	0_1	-1.834	-0.012	-0.016	-0.001	-0.000	-0.000	-0.000	-0.000	-0.000	-0.001
13	f	4_5	-1.830	-0.014	-0.021	-0.000	-0.000	-0.000	-0.000	-0.000	-0.000	-0.000
14	f	1_3	-1.830	-0.000	-0.014	-0.000	-0.000	-0.000	-0.000	-0.000	-0.000	-0.021
15	f	4_3	-1.830	-0.021	-0.014	-0.000	-0.000	-0.000	-0.000	-0.000	-0.000	-0.000
16	u	0_1	-1.830	-0.021	-0.014	-0.000	-0.000	-0.000	-0.000	-0.000	-0.000	-0.000
17	u	4_3	-1.830	-0.021	-0.014	-0.000	-0.000	-0.000	-0.000	-0.000	-0.000	-0.000
18	u	5_1	-1.829	-0.013	-0.022	-0.000	-0.000	-0.000	-0.000	-0.000	-0.000	-0.000
19	u	2_1	-1.830	-0.017	-0.021	-0.000	-0.000	0.000	-0.000	-0.000	-0.000	-0.000
20	f	4_0	-1.830	-0.000	-0.017	-0.000	-0.000	-0.000	-0.000	0.000	-0.000	-0.021
21	f	2_4	-1.829	-0.022	-0.013	-0.000	-0.000	-0.000	-0.000	-0.000	-0.000	-0.000
22	d	0_1	-1.834	-0.017	-0.013	-0.000	-0.000	-0.000	-0.000	-0.000	-0.000	-0.000
23	f	3_1	-1.832	-0.008	-0.020	-0.000	-0.000	-0.000	-0.000	-0.000	-0.000	-0.000
24	f	1_0	-1.832	-0.009	-0.018	-0.000	-0.000	-0.000	-0.000	-0.000	-0.000	-0.000
25	u	1_5	-1.833	-0.019	-0.009	-0.000	-0.000	-0.000	-0.000	-0.000	-0.000	-0.000
26	u	4_1	-1.829	-0.012	-0.021	-0.000	-0.000	-0.000	-0.000	-0.000	-0.000	-0.000
27	f	0_5	-1.839	-0.023	-0.023	-0.000	-0.000	-0.000	-0.000	-0.000	-0.000	-0.000
28	d	0_4	-1.830	-0.008	-0.037	-0.000	-0.000	-0.000	-0.000	0.000	-0.000	-0.001
29	u	3_4	-1.829	-0.038	-0.007	-0.000	-0.000	-0.000	-0.000	0.000	-0.001	-0.000
30	u	3_2	-1.829	-0.038	-0.007	-0.000	-0.000	-0.000	-0.000	0.000	-0.001	-0.000
31	f	0_3	-1.817	-0.000	-0.006	-0.000	-0.000	-0.000	-0.000	-0.000	-0.001	-0.000
32	f	5_1	-1.817	-0.000	-0.006	-0.000	-0.000	-0.000	-0.000	-0.000	-0.001	-0.000
33	d	5_4	-1.826	-0.040	-0.000	-0.000	-0.000	-0.000	-0.000	0.000	-0.000	-0.002
34	d	5_0	-1.827	-0.040	-0.000	-0.000	-0.000	-0.000	-0.000	0.000	-0.000	-0.002
35	d	2_0	-1.826	-0.002	-0.038	-0.000	-0.000	-0.000	-0.000	0.000	-0.001	-0.002
36	d	2_4	-1.826	-0.000	-0.041	-0.000	-0.000	-0.000	-0.000	0.000	-0.000	-0.002
37	u	3_0	-1.816	-0.008	-0.000	-0.000	-0.000	-0.000	-0.000	-0.000	-0.000	-0.001
38	u	5_0	-1.816	-0.008	-0.000	-0.000	-0.000	-0.000	-0.000	-0.000	-0.000	-0.001
39	u	5_3	-1.816	-0.008	-0.000	-0.000	-0.000	-0.000	-0.000	-0.000	-0.000	-0.001
40	u	2_0	-1.825	-0.039	-0.001	-0.000	-0.000	-0.000	-0.000	0.000	-0.002	-0.001
41	u	2_4	-1.825	-0.001	-0.039	-0.000	-0.000	-0.000	-0.000	0.000	-0.002	-0.001
42	f	0_4	-1.826	-0.002	-0.038	-0.000	-0.000	-0.000	-0.000	0.000	-0.001	-0.002
43	u	1_0	-1.818	-0.009	-0.000	-0.000	-0.000	0.000	0.000	-0.000	-0.000	-0.001
44	f	3_0	-1.826	-0.038	-0.002	-0.000	-0.000	-0.000	-0.000	0.000	-0.001	-0.002
45	u	3_1	-1.826	-0.038	-0.002	-0.000	-0.000	-0.001	0.000	0.000	-0.002	0.000
46	f	2_1	-1.817	-0.000	-0.006	0.000	-0.000	0.000	-0.000	-0.000	-0.001	-0.000
47	f	2_5	-1.833	-0.015	-0.013	-0.000	-0.000	-0.000	-0.000	-0.000	-0.000	-0.000
48	f	2_0	-1.826	-0.001	-0.039	-0.000	-0.000	-0.000	-0.000	0.000	-0.001	-0.002
49	f	3_4	-1.839	-0.011	-0.017	-0.001	-0.000	-0.000	-0.001	0.000	-0.000	-0.000
50	u	2_3	-1.816	-0.008	-0.000	-0.000	-0.000	-0.000	-0.000	-0.000	-0.000	-0.001

Table S7: Charge transfer for the surface nickel, fluorine and the adsorbates for group I.

#	Orientation	Position	Ni	F	F	C	H	H	H	H	F	H
1	d	5_1	0.273	0.448	0.465	-0.548	-0.028	-0.044	-0.032	-0.705	-0.141	0.047
2	u	2_5	0.269	0.458	0.478	-0.552	-0.053	-0.032	-0.011	-0.714	-0.144	0.035
3	f	4_1	0.269	0.459	0.472	-0.559	-0.011	-0.049	-0.038	-0.716	-0.136	0.041
4	u	0_4	0.270	0.460	0.481	-0.552	-0.043	-0.044	-0.016	-0.716	-0.137	0.031

Continued on next page

#	Orientation	Position	Ni	F	F	C	H	H	H	H	F	H
5	d	4_0	0.269	0.467	0.365	-0.445	-0.721	-0.097	-0.085	-0.703	0.000	0.701
6	u	4_2	0.271	0.350	0.485	-0.465	-0.064	-0.080	-0.075	-0.701	-0.020	0.051
7	f	4_2	0.271	0.466	0.369	-0.492	-0.066	-0.052	-0.073	-0.708	-0.013	0.051
8	u	4_0	0.280	0.452	0.466	-0.543	-0.034	-0.055	-0.016	-0.712	-0.144	0.034
9	u	0_5	0.270	0.351	0.493	-0.463	-0.744	-0.073	-0.059	-0.083	0.022	0.048
10	f	3_5	0.265	0.476	0.373	-0.465	-0.092	-0.072	-0.052	-0.721	0.000	0.046
11	d	4_1	0.276	0.478	0.472	-0.490	-0.085	-0.026	-0.711	-0.087	-0.132	0.047
12	f	0_1	0.267	0.351	0.493	-0.472	-0.044	-0.079	-0.081	-0.719	-0.004	0.048
13	f	4_5	0.283	0.471	0.495	-0.493	-0.089	-0.038	-0.730	-0.067	-0.132	0.048
14	f	1_3	0.284	0.366	0.492	-0.477	-0.733	-0.094	-0.039	-0.071	-0.026	0.048
15	f	4_3	0.283	0.477	0.493	-0.492	-0.051	-0.091	-0.043	-0.733	-0.141	0.047
16	u	0_1	0.285	0.478	0.485	-0.465	-0.076	-0.096	-0.041	-0.724	-0.141	0.047
17	u	4_3	0.285	0.479	0.491	-0.470	-0.099	-0.076	-0.043	-0.731	-0.131	0.046
18	u	5_1	0.284	0.469	0.495	-0.512	-0.060	-0.010	-0.092	-0.707	-0.140	0.022
19	u	2_1	0.282	0.462	0.484	-0.544	-0.045	-0.735	-0.019	-0.041	-0.142	0.043
20	f	4_0	0.282	0.362	0.479	-0.562	-0.022	-0.017	-0.048	-0.736	-0.036	0.043
21	f	2_4	0.286	0.482	0.488	-0.508	-0.003	-0.094	-0.060	-0.712	-0.148	0.019
22	d	0_1	0.266	0.462	0.495	-0.549	-0.040	-0.031	-0.733	-0.030	-0.142	0.056
23	f	3_1	0.268	0.484	0.496	-0.477	-0.060	-0.734	-0.034	-0.103	-0.140	0.048
24	f	1_0	0.272	0.474	0.502	-0.507	-0.095	-0.706	-0.029	-0.043	-0.142	0.029
25	u	1_5	0.265	0.477	0.522	-0.532	0.005	-0.042	-0.091	-0.755	-0.152	0.045
26	u	4_1	0.285	0.466	0.498	-0.504	-0.125	-0.010	-0.037	-0.723	-0.140	0.046
27	f	0_5	0.274	0.449	0.476	-0.558	-0.043	-0.019	-0.709	-0.032	-0.139	0.038
28	d	0_4	0.284	0.357	0.478	-0.419	-0.097	-0.101	-0.083	-0.737	0.012	0.037
29	u	3_4	0.286	0.459	0.375	-0.394	-0.115	-0.126	-0.065	-0.735	0.011	0.039
30	u	3_2	0.285	0.457	0.375	-0.402	-0.105	-0.082	-0.112	-0.731	-0.000	0.052
31	f	0_3	0.290	0.345	0.522	-0.437	-0.719	-0.080	-0.027	-0.126	-0.031	0.024
32	f	5_1	0.290	0.346	0.521	-0.422	-0.081	-0.140	-0.028	-0.716	-0.034	0.025
33	d	5_4	0.292	0.452	0.371	-0.437	-0.070	-0.078	-0.111	-0.731	0.014	0.033
34	d	5_0	0.294	0.453	0.370	-0.414	-0.083	-0.097	-0.103	-0.723	0.004	0.043
35	d	2_0	0.293	0.359	0.474	-0.402	-0.084	-0.093	-0.129	-0.723	0.003	0.042
36	d	2_4	0.294	0.350	0.470	-0.426	-0.100	-0.061	-0.112	-0.717	0.006	0.040
37	u	3_0	0.291	0.487	0.364	-0.413	-0.079	-0.721	-0.056	-0.123	-0.028	0.035
38	u	5_0	0.291	0.489	0.363	-0.421	-0.031	-0.713	-0.080	-0.138	-0.036	0.033
39	u	5_3	0.294	0.495	0.365	-0.440	-0.017	-0.722	-0.089	-0.127	-0.026	0.027
40	u	2_0	0.297	0.455	0.375	-0.388	-0.102	-0.121	-0.094	-0.720	0.006	0.039
41	u	2_4	0.297	0.358	0.473	-0.445	-0.080	-0.068	-0.114	-0.722	-0.007	0.053
42	f	0_4	0.296	0.361	0.474	-0.399	-0.117	-0.071	-0.119	-0.727	0.008	0.038
43	u	1_0	0.276	0.504	0.370	-0.426	-0.112	-0.090	-0.051	-0.719	-0.031	0.029
44	f	3_0	0.296	0.455	0.381	-0.404	-0.092	-0.120	-0.092	-0.727	0.005	0.041
45	u	3_1	0.295	0.459	0.376	-0.398	-0.093	-0.707	-0.117	-0.100	0.002	0.027
46	f	2_1	0.295	0.358	0.505	-0.405	-0.068	-0.709	-0.077	-0.134	-0.029	0.024
47	f	2_5	0.266	0.468	0.488	-0.560	-0.048	-0.032	-0.012	-0.729	-0.139	0.050
48	f	2_0	0.297	0.359	0.473	-0.396	-0.116	-0.082	-0.114	-0.721	0.011	0.035
49	f	3_4	0.265	0.363	0.494	-0.502	-0.696	-0.059	-0.093	-0.034	-0.026	0.045
50	u	2_3	0.291	0.490	0.363	-0.425	-0.031	-0.715	-0.079	-0.135	-0.034	0.032

Table S8: Calculated frequencies for group I.

5_1	2_5	4_1	0_4	4_0	4_2	4_2	4_0	0_5	3_5
d	u	f	u	d	u	f	u	u	f
3089.73	3081.22	3077.65	3080.85	3461.13	3528.24	3532.31	3073.98	3531.43	3523.9
3073.32	3072.65	3076.32	3074.14	3142.96	3151.08	3152.18	3067.07	3144.37	3145.03
2986.91	2984.12	2985.43	2984.23	3139.72	3130.35	3131.97	3062.1	3129.33	3129.57
2587.98	2618.35	2627.06	2632.45	3027.53	3024.26	3025.96	2978.88	3022.39	3022.93
2570.81	2601.52	2613.08	2598.5	1614.71	1683.56	1686.79	2861.36	1796.87	1804.49
1442.52	1448.9	1444.29	1443.77	1456.99	1455.24	1452.67	1448.33	1455.89	1456.48

Continued on next page

1437.12	1442.98	1439.98	1440.14	1425.75	1437.8	1436.27	1441.81	1433.89	1434.79
1424.84	1427.97	1430.01	1432.3	1421.7	1410.74	1410.59	1431.12	1412.93	1413.69
1141.56	1151.39	1151.53	1150.49	1271.45	1293.37	1292.14	1156.36	1278.34	1277.01
1136.39	1144.41	1140.02	1146.09	1150.92	1154.78	1153.54	1146.5	1156.46	1157.24
1131.53	1128.3	1133.16	1134.15	1141.23	1135.35	1135.02	1100.89	1135.63	1135.94
1075.45	1069.75	1064.36	1066.39	1098.87	1078.15	1078.45	970.87	1059.83	1059.18
1024.66	1020.52	1025.84	1021.89	897.38	893.94	894.45	960.86	922.95	923.52
976.49	966.65	972.01	969.41	782.8	749.07	748.6	822.52	747.73	751.16
876.57	866.87	865.9	866.72	679.23	663.99	665.38	733.24	662.88	668.43
328.08	326.01	322.79	322.99	446.45	426.12	427.11	328.06	647.46	645.81
306.51	303.74	301.04	301.01	332.0	320.43	321.36	288.01	412.62	410.63
248.05	241.28	241.59	241.57	221.75	224.66	223.7	237.08	228.54	228.74
243.71	237.92	238.22	237.73	178.03	197.16	195.35	198.26	184.07	187.31
166.43	170.58	173.6	170.05	165.92	167.51	161.71	146.55	147.83	150.08
148.7	150.92	155.79	151.91	153.78	151.58	143.82	138.26	144.12	146.42
85.5	94.98	95.12	86.02	141.04	132.95	133.62	120.66	125.43	124.62
57.2	75.16	66.47	51.7	117.57	117.27	116.2	83.71	108.68	114.3
30.26	71.52	61.1	36.33	106.33	102.14	103.53	48.02	93.52	96.13
17.8	50.34	31.69	8.83	99.06	99.91	98.16	27.84i	92.09	89.1
33.35i	33.02	15.19	16.33i	49.18	56.58	63.69	42.83i	45.25	44.9
53.58i	29.45i	35.85i	65.41i	37.03	35.89	49.94	50.5i	40.5	43.09
4_1	0_1	4_5	1_3	4_3	0_1	4_3	5_1	2_1	4_0
d	f	f	f	f	u	u	u	u	f
3391.67	3486.39	3388.38	3392.74	3391.02	3388.9	3395.01	3449.64	3428.95	3444.21
3137.25	3142.97	3131.38	3133.82	3135.03	3137.31	3140.03	3146.17	3082.03	3084.25
3122.5	3135.93	3116.49	3119.58	3121.75	3122.04	3124.03	3111.75	3069.23	3070.18
3016.36	3026.87	3011.08	3013.8	3015.79	3016.2	3018.27	3012.13	2982.54	2983.38
1961.57	1780.29	2014.40	1999.8	2008.45	1998.13	1991.19	2025.79	1882.29	1904.96
1452.62	1457.36	1453.93	1455.17	1454.55	1454.99	1452.85	1444.9	1448.56	1448.94
1436.43	1426.07	1442.39	1438.36	1437.61	1436.47	1432.13	1427.62	1442.02	1438.22
1415.96	1420.41	1417.26	1417.07	1416.34	1415.73	1416.22	1425.45	1425.7	1428.45
1237.47	1259.07	1229.53	1232.59	1231.11	1233.05	1233.96	1221.9	1191.13	1196.48
1161.73	1151.91	1164.73	1162.27	1161.45	1160.55	1157.08	1149.03	1149.8	1153.96
1140.98	1141.46	1138.89	1140.36	1139.94	1139.97	1143.71	1146.12	1144.86	1145.04
1054.74	1082.55	1047.49	1049.69	1047.35	1049.2	1049.54	1047.31	1099.01	1089.72
911.25	928.67	912.24	912.71	912.52	912.58	911.72	911.99	968.77	966.98
861.17	761.85	859.04	857.28	858.89	862.42	862.12	797.6	759.98	740.26
605.08	670.06	599.74	601.14	601.03	604.9	606.24	616.43	618.26	608.47
382.25	650.43	371.97	372.69	371.72	371.33	372.18	378.37	363.58	366.16
312.0	422.63	309.29	309.43	308.74	312.85	315.55	272.43	249.73	239.82
262.85	227.09	250.49	252.69	251.06	252.49	253.38	265.48	231.33	225.99
223.52	183.13	217.45	216.76	217.58	217.72	221.45	214.88	173.43	167.74
167.44	168.81	160.05	159.24	160.14	160.49	160.32	153.98	160.63	157.71
133.14	150.35	138.87	144.65	137.44	138.91	145.19	147.44	127.84	131.9
102.78	125.38	102.74	106.41	103.32	107.61	116.34	138.4	124.0	112.73
98.18	111.71	99.62	99.46	96.06	96.89	104.92	101.65	73.61	94.55
70.72	106.23	88.51	89.72	82.36	89.86	90.02	91.11	61.54	53.82

Continued on next page

62.45	91.07	63.12	75.53	70.83	71.41	73.7	65.92	50.94	43.73
54.93	54.18	47.57	53.36	50.89	54.17	58.07	28.34	11.76i	22.48
19.32i	48.59	36.80	37.03	32.64	10.7	35.82	20.9	17.7i	13.26i
2_4	0_1	3_1	1_0	1_5	4_1	0_5	0_4	3_4	3_2
f	d	f	f	u	u	f	d	u	u
3520.59	3200.51	3485.21	3471.29	3566.13	3462.73	3075.6	3185.38	3183.89	3186.33
3127.49	3081.46	3144.79	3126.14	3125.97	3138.16	3072.16	3161.63	3162.55	3172.11
3103.8	3078.21	3108.36	3114.69	3088.08	3103.99	2982.3	3046.03	3045.42	3051.71
3006.94	2986.92	3010.85	3008.76	2998.98	3006.6	2608.66	2896.88	2886.41	2871.84
2123.33	1806.95	2140.16	2084.23	2229.21	2042.25	2597.68	2594.06	2513.93	2546.5
1454.77	1440.05	1442.61	1456.16	1445.0	1443.86	1442.18	1452.51	1451.89	1450.2
1433.01	1436.82	1430.06	1430.8	1430.04	1431.57	1440.65	1423.94	1420.37	1416.94
1423.52	1426.77	1424.63	1419.62	1422.81	1428.49	1429.38	1407.51	1405.43	1405.15
1215.51	1281.66	1227.52	1210.14	1191.03	1230.83	1150.13	1158.73	1160.07	1155.53
1151.69	1148.08	1148.92	1151.34	1154.07	1153.46	1143.55	1128.07	1127.23	1127.39
1140.34	1141.24	1139.18	1148.48	1142.59	1144.75	1134.36	1058.55	1072.29	1060.15
1021.34	1124.35	1015.51	1018.31	1005.58	1022.75	1071.08	961.35	959.1	960.75
923.31	965.38	902.38	906.45	944.34	901.15	1023.12	944.21	947.99	953.38
707.54	896.31	793.68	847.54	677.69	848.59	970.09	822.68	832.31	828.55
555.07	704.43	632.93	650.97	630.61	578.88	868.97	788.33	790.49	785.31
357.14	628.63	593.83	607.77	541.8	371.73	321.81	522.59	520.7	523.14
278.54	384.28	345.18	360.47	348.25	272.62	298.83	301.38	298.72	304.03
246.52	245.09	234.15	261.01	253.94	246.26	243.96	267.17	273.64	273.03
188.94	181.37	198.37	173.13	162.41	204.77	233.08	202.04	204.53	207.0
156.89	130.19	156.21	152.7	147.07	160.91	171.23	177.67	166.56	181.62
133.02	100.59	145.2	136.95	130.88	136.49	154.95	156.56	157.36	163.41
107.75	92.3	128.09	115.68	100.31	126.79	87.71	151.08	152.34	157.56
79.48	52.9	106.37	102.18	95.89	87.24	54.48	130.76	128.78	132.93
71.77	46.21	85.87	85.55	58.74	76.96	46.41	122.16	116.73	121.81
68.49	21.17i	68.16	44.78	41.42	45.25	22.45	110.65	108.19	112.56
43.96	24.5i	27.01	37.55	35.37	6.35	21.73	59.83	70.29	54.63
30.45i	64.6i	25.33	34.76i	47.45i	36.9i	31.43i	41.38	32.81	12.56
0_3	5_1	5_4	5_0	2_0	2_4	3_0	5_0	5_3	2_0
f	f	d	d	d	d	u	u	u	u
3185.08	3186.67	3174.36	3169.59	3183.81	3171.02	3188.72	3189.9	3184.98	3184.96
3140.92	3141.66	3159.51	3157.83	3158.62	3160.34	3141.84	3142.69	3143.31	3162.93
3100.34	3097.6	3044.69	3041.71	3058.59	3048.75	3059.68	3067.27	3059.66	3067.5
3027.22	3027.89	3009.41	3001.66	3037.07	3027.5	3024.33	3026.3	3024.45	3042.57
2757.4	2749.28	2242.75	2239.05	2317.39	2210.46	2704.57	2712.48	2705.57	2235.11
1458.11	1457.96	1450.59	1454.05	1454.49	1453.63	1456.49	1457.31	1456.42	1452.93
1432.2	1432.1	1418.83	1418.89	1406.61	1414.06	1434.72	1434.24	1434.66	1405.91
1395.33	1394.48	1407.77	1406.71	1402.51	1405.5	1393.01	1392.32	1395.72	1400.38
1183.47	1185.07	1191.53	1191.28	1159.06	1195.31	1197.19	1196.27	1195.13	1161.72
1137.76	1136.73	1138.43	1140.94	1129.68	1140.22	1133.92	1134.77	1135.59	1128.54
1113.61	1113.17	1125.11	1125.66	1074.67	1125.16	1111.38	1110.55	1111.48	1081.86
887.41	888.86	942.07	943.44	983.89	932.5	902.69	902.98	900.23	1006.0
850.37	850.93	914.29	917.59	940.5	923.76	857.4	852.83	856.5	933.5

Continued on next page

832.72	834.81	908.9	909.78	898.73	909.58	837.21	834.09	836.11	893.47
802.45	803.36	824.37	824.22	793.98	824.23	801.26	799.87	798.44	796.19
478.86	482.46	497.1	496.77	492.96	491.26	433.09	431.09	428.69	494.01
371.11	373.46	308.88	309.16	310.83	313.9	329.74	327.55	324.79	302.76
272.21	274.85	294.58	294.51	288.53	298.55	241.43	239.55	240.74	290.89
221.95	221.82	171.24	169.06	166.08	168.24	205.51	204.09	197.24	169.96
174.87	173.01	144.94	141.9	138.21	142.59	158.61	158.32	152.85	141.98
143.29	139.84	134.43	133.49	125.09	135.14	149.35	146.12	149.94	122.04
117.57	118.09	115.1	110.84	114.06	112.8	123.46	120.48	115.18	112.38
99.97	98.13	96.21	100.32	100.43	89.56	107.23	101.48	99.25	102.16
79.65	83.24	82.45	81.08	96.79	83.21	83.34	83.3	77.49	88.93
59.97	57.59	62.72	69.05	79.04	73.83	60.03	56.78	60.38	79.75
42.14	41.18	41.75	32.19	45.31	48.27	42.71	41.36	17.84	46.38
27.58	18.55	15.03i	13.56	27.2	27.7	33.2	22.69	23.79i	34.73
2_4	0_4	1_0	3_0	3_1	2_1	2_5	2_0	3_4	2_3
u	f	u	f	u	f	f	f	f	u
3184.57	3185.65	3204.34	3186.61	3195.5	3754.14	3283.45	3185.49	3554.09	3187.22
3161.93	3161.59	3168.14	3161.42	3185.64	3289.09	3084.86	3162.37	3149.42	3140.8
3065.76	3049.32	3042.63	3050.64	3143.29	3150.62	3075.37	3064.57	3140.76	3067.3
3041.76	3026.85	3007.01	3029.34	3059.36	3132.33	2986.6	3041.98	3031.48	3024.32
2239.49	2295.64	2638.24	2287.97	2252.54	3015.59	1924.03	2242.91	1788.21	2712.82
1453.16	1455.11	1439.52	1453.38	1439.3	1461.47	1445.22	1453.9	1440.97	1456.79
1406.29	1404.03	1429.13	1404.04	1410.98	1453.91	1438.72	1407.24	1432.93	1433.35
1401.55	1403.14	1393.75	1401.36	1395.34	1412.38	1427.48	1399.86	1409.77	1392.76
1161.38	1160.65	1195.33	1162.25	1154.55	1154.43	1267.55	1162.34	1270.45	1195.81
1127.67	1126.39	1120.18	1125.72	1129.18	1111.35	1151.59	1129.86	1147.76	1135.45
1081.45	1079.88	1116.84	1080.71	1089.07	844.8	1143.6	1084.55	1133.03	1111.29
1003.79	986.39	865.12	989.98	998.13	815.59	1107.18	999.75	1059.92	902.28
935.4	948.86	855.7	945.9	925.09	725.15	966.18	934.64	935.58	851.49
894.75	907.0	844.98	905.61	865.76	574.03	869.45	896.58	742.37	832.8
796.42	790.95	810.23	789.12	787.87	523.84	665.65	797.24	666.77	798.49
493.77	493.51	459.7	493.09	487.23	422.4	569.15	494.96	631.36	431.5
300.54	307.05	341.68	305.35	304.7	253.96	368.36	301.76	422.16	327.48
287.41	288.7	235.31	290.68	294.25	193.13	223.14	288.99	224.41	238.14
164.2	164.13	204.41	164.39	163.84	174.23	180.56	166.23	172.3	203.34
139.55	144.08	147.09	148.92	151.94	141.55	129.2	141.09	139.5	157.42
124.42	129.87	132.22	131.0	123.78	115.93	104.16	119.96	132.29	144.35
120.63	114.33	124.45	109.11	106.21	98.47	97.48	109.22	120.07	120.15
98.53	104.03	92.96	96.52	96.54	83.51	62.04	100.16	102.3	103.2
87.69	94.5	60.34	86.21	77.64	74.27	54.48	85.29	91.62	82.09
73.77	74.87	44.92	71.01	41.17	47.65	49.36	76.49	58.77	53.45
28.91	43.15	34.4	52.8	31.77	26.72	33.91	46.5	37.14	38.52
15.79	22.75	33.58i	27.76	137.59i	7.37i	32.03i	21.37	34.24i	21.94

2.2 Group 2

Table S9: Adsorption energies and bond distances for the adsorbed structures in group II.

#	Orientation	Position	E_{ads}	d(CH)	d(CH)	d(CF)	d(CF)	d(NiF)	d(NiF)	d(HF)	d(HH)
1	u	1_4	-9.1177	1.092	1.094	1.369	1.421	2.063	2.194	1.047	0.752
2	u	0_3	-9.0037	1.094	1.095	1.370	1.416	2.176	2.355	1.039	0.752
3	d	2_1	-8.9944	1.093	1.096	1.370	1.417	2.043	2.222	1.041	0.751
4	f	1_5	-7.0577	1.095	1.095	1.357	1.452	1.830	2.860	1.010	0.752

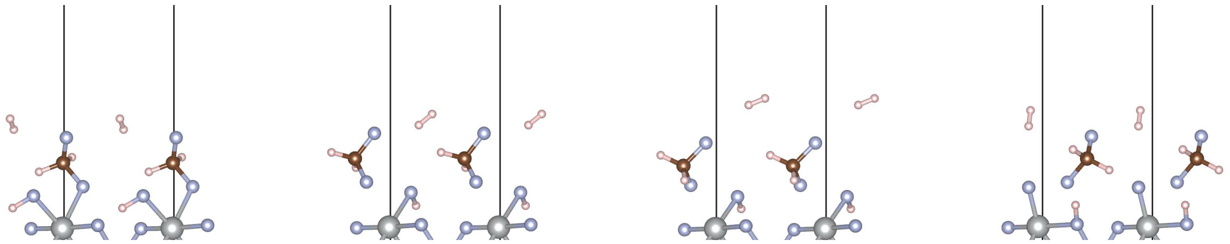

(1) u 1_4 (2) u 0_3 (3) d 2_1 (4) f 1_5

Figure S3: Adsorbed structures belonging to group II.

Table S10: Magnetic moments on the surface nickel, fluorine and on the adsorbates for group II.

#	Orientation	Position	Ni	F	F	C	H	H	H	H	F	H
1	u	1_4	-1.827	-0.012	-0.022	-0.000	-0.000	-0.000	-0.000	-0.000	-0.000	-0.000
2	u	0_3	-1.830	-0.000	-0.009	-0.000	-0.000	-0.000	-0.000	-0.000	-0.019	-0.000
3	d	2_1	-1.829	-0.023	-0.009	-0.001	-0.000	-0.000	-0.000	-0.000	-0.000	-0.001
4	f	1_5	-1.816	-0.002	-0.059	0.000	0.000	0.000	0.000	-0.000	-0.000	-0.000

Table S11: Charge transfer for the surface nickel, fluorine and the adsorbates for group II.

#	Orientation	Position	Ni	F	F	C	H	H	H	H	F	H
1	u	1_4	0.282	0.343	0.494	-1.099	-0.071	0.022	0.007	-0.113	-0.157	0.041
2	u	0_3	0.274	0.348	0.362	-1.056	-0.715	0.011	0.018	-0.108	-0.020	0.638
3	d	2_1	0.274	0.464	0.372	-1.063	-0.005	-0.086	0.036	-0.140	-0.152	0.044
4	f	1_5	0.319	0.347	0.465	-1.031	-0.726	-0.167	-0.104	-0.002	-0.148	0.783

Table S12: Calculated frequencies for group II

1_4	0_3	2_1	1_5
u	u	d	f
4270.63	4273.81	4302.96	4264.3
3151.92	3139.79	3140.79	3137.66
3052.38	3042.85	3038.53	3033.42
2131.67	2238.94	2210.16	2714.56
1451.06	1452.43	1470.87	1473.6
1383.83	1388.61	1387.76	1388.07
1212.99	1216.33	1215.0	1216.32

Continued on next page

1194.47	1180.37	1194.0	1115.41
1125.26	1127.56	1135.43	1099.02
1073.81	1069.66	1066.55	927.25
1032.24	1012.73	1031.55	874.22
919.97	938.65	920.71	813.03
495.82	625.8	491.26	484.92
363.82	473.96	359.08	463.12
253.95	326.19	259.11	321.7
241.48	232.99	223.12	257.34
224.24	197.63	183.91	240.91
215.33	189.68	171.91	179.29
169.7	157.01	162.35	171.52
153.67	143.45	155.13	130.42
146.55	138.2	132.18	112.89
116.38	91.74	108.57	104.52
95.77	75.79	93.5	77.13
84.91	66.75	85.48	65.25
74.97	59.15	62.41	41.9
60.02	28.38	51.6	10.56
42.83	17.01i	37.84	19.9i

2.3 Group 3

Table S13: Adsorption energies and bond distances for the adsorbed structures in group III.

#	Orientation	Position	E_{ads}	d(CH)	d(CH)	d(CH)	d(NiF)	d(NiF)	d(HF)	d(HF)
1	u	5_4	-3.2646	1.089	1.089	1.092	1.775	2.063	0.976	0.994
2	f	5_0	-3.2623	1.089	1.089	1.091	1.774	2.061	0.976	0.995
3	f	5_4	-3.2569	1.089	1.089	1.092	1.774	2.060	0.975	0.992
4	f	1_4	-2.5749	1.089	1.089	1.092	1.774	2.062	0.976	0.995
5	u	3_5	-2.3095	1.089	1.090	1.093	1.777	2.066	0.975	1.013

Table S14: Magnetic moments on the surface nickel, fluorine and on the adsorbates for group III.

#	Orientation	Position	Ni	F	F	C	H	H	H	H	F	H
1	u	5_4	-2.227	-0.039	-0.237	0.264	0.027	-0.004	-0.004	-0.005	0.070	-0.000
2	f	5_0	-2.227	-0.039	-0.237	0.263	0.027	-0.004	-0.004	-0.004	-0.000	0.072
3	f	5_4	-2.230	-0.245	-0.040	-0.268	0.004	-0.026	0.004	0.005	-0.000	-0.072
4	f	1_4	-2.227	-0.236	-0.039	0.263	-0.004	0.027	-0.004	-0.005	-0.000	0.071
5	u	3_5	-2.223	-0.039	-0.238	-0.005	-0.000	0.000	-0.000	-0.001	-0.002	-0.000

Table S15: Charge transfer for the surface nickel, fluorine and the adsorbates for group III.

#	Orientation	Position	Ni	F	F	C	H	H	H	H	F	H
1	u	5_4	0.002	0.439	0.323	0.153	-0.641	-0.078	-0.033	-0.165	-0.046	0.032

Continued on next page

#	Orientation	Position	Ni	F	F	C	H	H	H	H	F	H
2	f	5_0	0.002	0.453	0.323	0.151	-0.622	-0.067	-0.051	-0.159	-0.063	0.019
3	f	5_4	-0.000	0.304	0.469	0.116	-0.045	-0.627	-0.044	-0.143	-0.062	0.021
4	f	1_4	0.002	0.305	0.470	0.141	-0.069	-0.625	-0.051	-0.146	-0.060	0.020
5	u	3_5	0.004	0.452	0.323	0.116	-0.576	-0.050	-0.071	-0.146	-0.088	0.022

Table S16: Calculated frequencies for group III

5_4	5_0	5_4	1_4	3_5
u	f	f	f	u
3276.97	3271.02	3284.45	3277.16	3258.19
3204.32	3206.17	3202.34	3204.76	3204.36
3189.74	3195.09	3188.03	3191.48	3183.48
3017.98	3022.37	3017.04	3019.4	3007.71
2830.64	2805.98	2856.17	2813.42	2465.13
1369.46	1368.04	1372.91	1369.91	1367.38
1357.21	1357.41	1354.8	1356.85	1357.61
1000.55	1009.57	996.72	1006.71	992.86
839.86	842.76	832.14	840.47	901.01
771.59	774.57	774.66	772.67	754.87
648.19	658.83	638.79	655.96	678.62
582.51	585.1	577.22	583.72	602.88
495.49	496.65	498.7	495.93	480.24
337.59	338.06	339.13	337.8	380.35
315.4	316.93	308.61	314.84	333.22
259.51	268.36	264.1	265.46	270.35
246.91	250.34	252.24	250.78	238.99
240.45	241.89	239.56	242.48	219.14
175.04	177.56	179.38	176.54	177.41
172.26	171.49	177.39	171.67	161.06
143.75	141.14	148.53	142.46	140.88
137.77	139.91	138.71	137.68	131.28
115.57	117.39	115.43	117.3	108.93
93.12	93.78	102.06	96.17	98.88
75.36	76.26	92.44	77.79	57.79
43.68	50.79	55.54	50.04	40.99
24.43	42.36	27.29	39.03	4.48

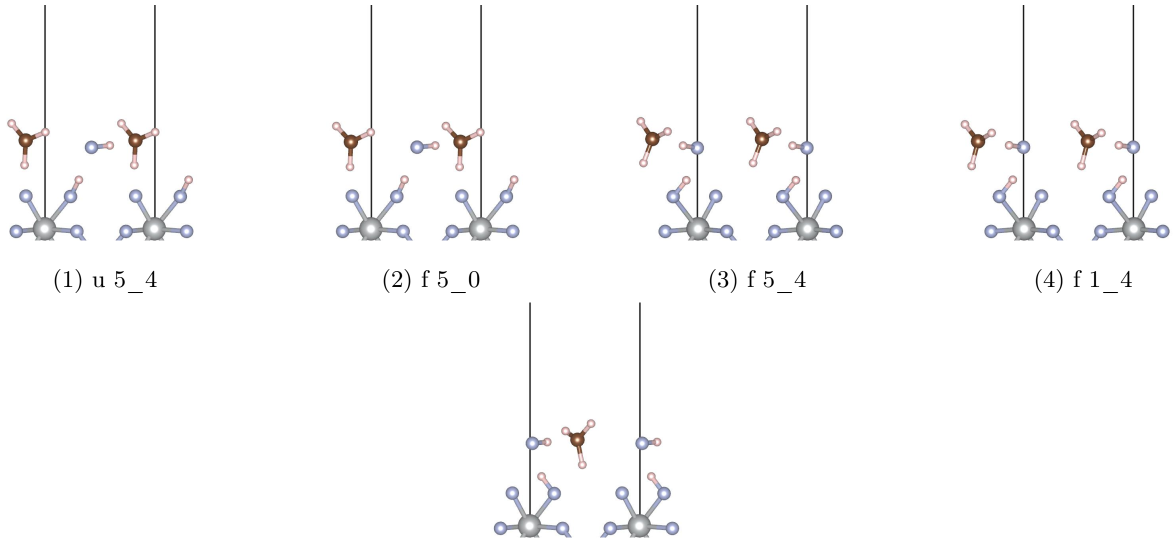

(1) u 5_4 (2) f 5_0 (3) f 5_4 (4) f 1_4

(5) u 3_5

Figure S4: Adsorbed structures belonging to group III.

2.4 Group 4

Table S17: Adsorption energies and bond distances for the adsorbed structures in group IV.

#	Orientation	Position	E_{ads}	d(CH)	d(CH)	d(CH)	d(CH)	d(NiF)	d(NiF)	d(HF)
1	d	1_5	-1.4193	1.096	1.096	1.096	1.097	1.837	1.930	1.076
2	f	5_3	-1.3821	1.096	1.096	1.097	1.097	1.836	1.945	1.058
3	u	0_2	-0.9983	1.095	1.096	1.099	1.099	1.705	1.710	0.950
4	f	3_2	-0.8739	1.095	1.095	1.096	1.111	1.779	1.926	0.977
5	d	2_5	-0.7621	1.095	1.095	1.096	1.110	1.839	1.843	0.972
6	d	1_2	-0.7281	1.095	1.096	1.099	1.099	1.866	1.896	0.949
7	f	5_2	-0.7279	1.095	1.096	1.099	1.099	1.870	1.892	0.948
8	d	5_2	-0.7272	1.095	1.096	1.099	1.099	1.876	1.885	0.949
9	d	4_2	-0.7155	1.095	1.096	1.099	1.099	1.874	1.889	0.948
10	d	0_2	-0.7058	1.095	1.096	1.099	1.099	1.875	1.887	0.948
11	d	1_3	-0.7049	1.095	1.096	1.098	1.099	1.872	1.889	0.948
12	d	2_3	-0.7027	1.096	1.098	1.098	1.100	1.874	1.881	0.949
13	d	5_3	-0.6988	1.096	1.096	1.100	1.100	1.866	1.889	0.949
14	f	2_3	-0.6937	1.096	1.096	1.099	1.099	1.866	1.889	0.949
15	d	1_4	-0.6909	1.094	1.096	1.100	1.100	1.868	1.887	0.949
16	d	4_3	-0.6859	1.095	1.096	1.099	1.099	1.875	1.888	0.948
17	d	3_2	-0.6856	1.095	1.096	1.098	1.098	1.834	1.941	0.948
18	d	3_5	-0.6842	1.096	1.096	1.099	1.100	1.863	1.894	0.949
19	d	3_4	-0.6829	1.096	1.098	1.098	1.100	1.876	1.881	0.949
20	u	4_5	-0.6797	1.095	1.096	1.098	1.099	1.866	1.899	0.947
21	d	0_3	-0.6742	1.095	1.096	1.099	1.099	1.879	1.883	0.948
22	d	3_0	-0.6705	1.095	1.098	1.099	1.100	1.874	1.882	0.949
23	d	1_0	-0.6703	1.096	1.096	1.099	1.100	1.867	1.889	0.949
24	u	1_2	-0.5754	1.095	1.096	1.099	1.100	1.814	1.919	0.951
25	f	1_2	-0.5744	1.095	1.096	1.099	1.099	1.815	1.920	0.950
26	u	5_2	-0.1752	1.095	1.096	1.099	1.100	1.781	1.994	0.950

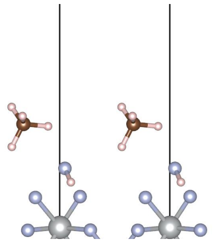
(1) d 1_5

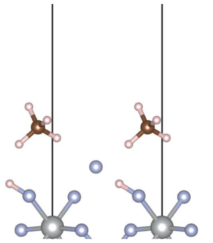
(2) f 5_3

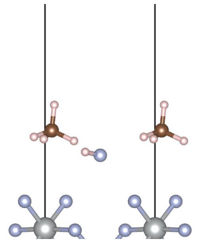
(3) u 0_2

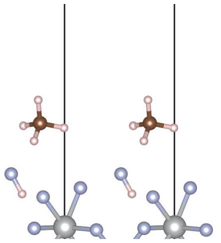
(4) f 3_2

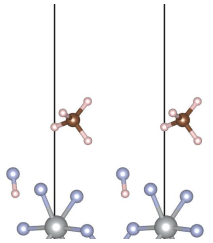
(5) d 2_5

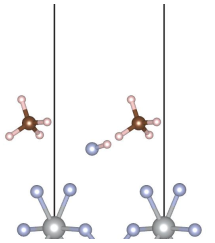
(6) d 1_2

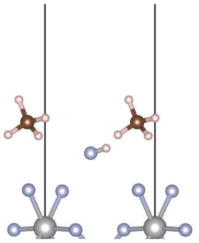
(7) f 5_2

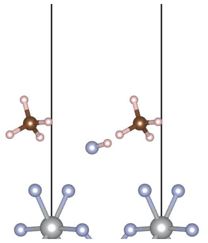
(8) d 5_2

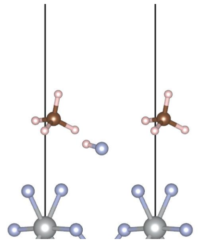
(9) d 4_2

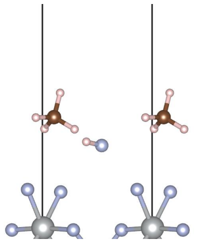
(10) d 0_2

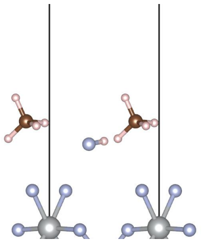
(11) d 1_3

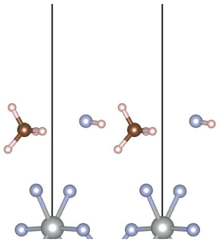
(12) d 2_3

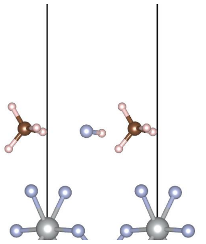
(13) d 5_3

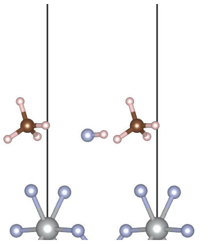
(14) f 2_3

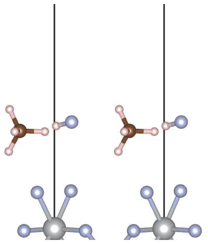
(15) d 1_4

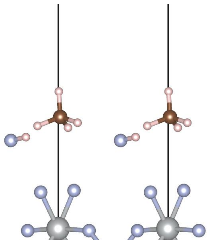
(16) d 4_3

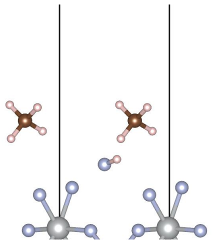

(17) d 3_2

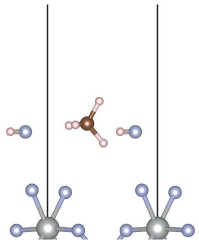

(18) d 3_5

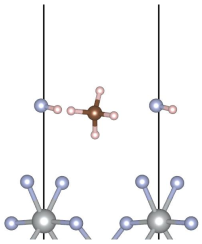

(19) d 3_4

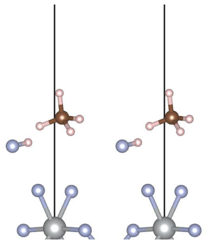

(20) u 4_5

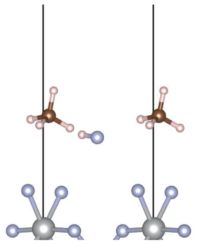

(21) d 0_3

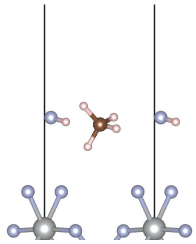

(22) d 3_0

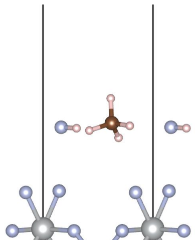

(23) d 1_0

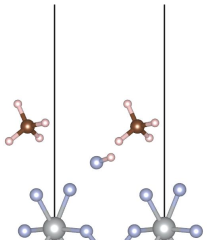

(24) u 1_2

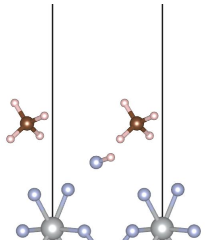

(25) f 1_2

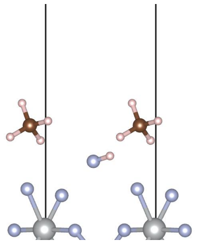

(26) u 5_2

Figure S5: Adsorbed structures belonging to group IV.

Table S18: Magnetic moments on the surface nickel, fluorine and on the adsorbates for group IV.

#	Orientation	Position	Ni	F	F	C	H	H	H	H	H	F
1	d	1_5	-2.240	-0.147	-0.581	-0.003	-0.001	-0.002	-0.000	-0.001	0.002	-0.397
2	f	5_3	-2.224	0.181	-0.065	0.003	0.002	0.001	0.000	0.003	-0.004	0.512
3	u	0_2	0.106	-0.020	-0.017	-0.000	-0.000	-0.000	-0.000	-0.000	-0.006	0.000
4	f	3_2	-2.212	-0.540	-0.425	-0.026	-0.003	-0.003	-0.002	-0.029	0.002	-0.226
5	d	2_5	-2.222	-0.448	-0.522	-0.024	0.002	-0.003	-0.002	-0.029	-0.003	-0.204
6	d	1_2	-2.219	-0.586	-0.577	-0.001	-0.000	-0.000	-0.000	-0.000	0.000	-0.025
7	f	5_2	-2.220	-0.575	-0.582	-0.001	-0.000	-0.000	-0.000	-0.000	0.000	-0.033
8	d	5_2	-2.220	-0.582	-0.585	-0.001	-0.000	0.000	-0.000	-0.000	0.000	-0.020
9	d	4_2	-2.219	-0.585	-0.579	-0.001	-0.000	-0.000	-0.000	-0.000	0.000	-0.026
10	d	0_2	-2.220	-0.571	-0.598	-0.001	-0.000	-0.000	-0.000	-0.000	-0.000	-0.017
11	d	1_3	-2.220	-0.586	-0.582	-0.001	-0.000	-0.000	-0.000	-0.000	0.000	-0.013
12	d	2_3	-2.221	-0.575	-0.574	-0.002	-0.000	-0.001	-0.001	-0.002	0.000	-0.002
13	d	5_3	-2.221	-0.571	-0.575	-0.002	-0.001	-0.000	-0.000	-0.001	0.000	-0.005
14	f	2_3	-2.220	-0.581	-0.578	-0.001	-0.000	-0.001	-0.000	-0.001	0.000	-0.006
15	d	1_4	-2.222	-0.571	-0.566	-0.004	-0.001	-0.001	-0.000	-0.003	0.000	-0.006
16	d	4_3	-2.219	-0.592	-0.580	-0.001	-0.000	-0.000	-0.001	-0.000	0.000	-0.012
17	d	3_2	-2.216	-0.575	-0.538	-0.001	-0.000	-0.000	-0.000	-0.001	0.001	-0.085
18	d	3_5	-2.219	-0.578	-0.581	-0.001	-0.000	-0.000	-0.001	-0.001	0.000	-0.004
19	d	3_4	-2.221	-0.570	-0.574	-0.001	-0.000	-0.001	-0.001	-0.001	0.000	-0.002
20	u	4_5	-2.219	-0.586	-0.577	-0.001	-0.000	-0.000	-0.000	-0.000	-0.021	0.000
21	d	0_3	-2.219	-0.580	-0.595	-0.000	-0.000	-0.000	-0.000	-0.000	0.000	-0.011
22	d	3_0	-2.221	-0.566	-0.572	-0.002	-0.000	-0.001	-0.001	-0.001	0.000	-0.004
23	d	1_0	-2.220	-0.577	-0.578	-0.001	-0.000	-0.000	-0.000	-0.000	0.000	-0.004
24	u	1_2	-2.228	0.394	0.156	0.000	0.000	0.000	-0.000	0.000	0.088	-0.001
25	f	1_2	-2.229	0.395	0.159	0.000	0.000	0.000	-0.000	0.000	-0.001	0.086
26	u	5_2	-0.908	-0.305	-0.601	-0.001	-0.000	-0.000	0.000	-0.000	-0.067	0.001

Table S19: Charge transfer for the surface nickel, fluorine and the adsorbates for group IV.

#	Orientation	Position	Ni	F	F	C	H	H	H	H	F	H
1	d	1_5	-0.015	0.353	0.030	-0.002	-0.013	-0.012	0.009	-0.001	-0.356	0.042
2	f	5_3	-0.011	0.067	0.433	-0.008	-0.015	0.007	-0.001	-0.008	-0.467	0.044
3	u	0_2	0.079	0.111	0.118	-0.003	-0.033	-0.009	0.013	-0.007	-0.058	0.045
4	f	3_2	0.018	0.051	0.162	-0.015	0.007	-0.030	-0.006	-0.065	-0.208	0.026
5	d	2_5	0.002	0.130	0.075	-0.033	-0.725	-0.014	0.013	-0.074	-0.193	0.753
6	d	1_2	0.001	0.006	0.030	0.025	-0.016	-0.021	-0.009	-0.014	-0.050	0.054
7	f	5_2	0.001	0.015	0.028	0.020	-0.027	-0.024	0.006	-0.008	-0.063	0.057
8	d	5_2	-0.002	0.004	0.026	0.028	-0.033	0.014	-0.020	-0.025	-0.054	0.063
9	d	4_2	0.003	0.004	0.032	-0.011	-0.007	-0.004	0.012	-0.023	-0.055	0.055
10	d	0_2	0.001	0.020	0.008	0.016	-0.033	-0.023	0.014	-0.007	-0.040	0.049
11	d	1_3	0.002	0.006	0.022	0.034	-0.009	-0.044	-0.004	-0.012	-0.039	0.051
12	d	2_3	-0.002	0.016	0.032	0.035	-0.041	-0.025	-0.005	-0.020	-0.050	0.060
13	d	5_3	-0.002	0.016	0.035	0.009	-0.020	-0.046	-0.015	0.017	-0.050	0.058
14	f	2_3	0.001	0.008	0.028	0.007	-0.029	-0.025	0.008	-0.008	-0.039	0.052
15	d	1_4	-0.002	0.020	0.040	-0.014	0.001	-0.010	-0.023	-0.018	-0.054	0.061
16	d	4_3	-0.001	-0.004	0.032	-0.012	-0.017	0.031	-0.014	-0.022	-0.038	0.052
17	d	3_2	0.001	0.027	0.070	0.007	-0.012	0.006	0.003	-0.031	-0.107	0.042
18	d	3_5	0.001	0.012	0.025	0.027	-0.034	-0.000	-0.018	-0.023	-0.045	0.059
19	d	3_4	0.000	0.016	0.033	0.037	-0.056	-0.013	-0.026	0.002	-0.048	0.053
20	u	4_5	0.003	-0.004	0.037	-0.007	0.011	-0.020	-0.011	-0.004	-0.046	0.050
21	d	0_3	0.003	0.008	0.013	-0.019	-0.014	-0.001	0.012	-0.014	-0.033	0.048
22	d	3_0	-0.001	0.020	0.036	0.068	-0.049	-0.056	-0.015	-0.007	-0.051	0.055
23	d	1_0	-0.001	0.014	0.027	0.058	-0.052	-0.014	-0.029	-0.012	-0.036	0.047
24	u	1_2	-0.007	0.035	0.095	0.002	-0.007	-0.016	0.003	-0.017	-0.110	0.043
25	f	1_2	-0.007	0.033	0.095	0.010	-0.014	-0.014	0.001	-0.015	-0.105	0.036
26	u	5_2	0.130	0.031	0.022	-0.031	-0.004	-0.019	0.021	-0.001	-0.087	0.042

Table S20: Calculated frequencies for group IV

1_5	5_3	0_2	3_2	2_5	1_2	5_2	5_2	4_2
d	f	u	f	d	d	f	d	d
3090.69	3090.42	3746.3	3175.63	3259.51	3755.63	3771.93	3756.69	3783.77
3087.94	3077.81	3102.66	3096.41	3089.96	3099.73	3098.14	3097.84	3105.72
3072.56	3069.25	3071.0	3091.79	3086.13	3071.06	3070.82	3072.42	3072.68
2973.01	2970.44	3064.95	3011.41	3008.88	3063.3	3062.73	3064.2	3063.94
1646.8	1827.24	2960.68	2831.56	2843.62	2957.05	2956.98	2957.25	2959.92
1484.59	1492.51	1515.05	1397.12	1386.17	1527.53	1527.28	1528.78	1519.79
1477.87	1470.65	1512.71	1370.06	1363.46	1518.85	1521.11	1518.81	1519.31
1279.13	1276.87	1320.06	1209.18	1197.12	1304.97	1301.1	1304.09	1311.24
1272.47	1265.33	1299.18	1151.12	1126.97	1294.07	1292.1	1293.82	1296.58
1268.23	1260.79	1253.69	1144.58	1097.36	1271.89	1274.88	1273.45	1265.0
1115.06	1119.92	571.5	750.64	698.88	469.68	468.68	469.0	471.79
935.26	963.47	552.56	637.59	606.07	433.93	417.08	427.9	411.45
497.38	495.63	406.0	502.05	476.95	341.01	325.7	339.88	345.99
382.51	430.11	366.23	306.98	257.07	260.27	259.42	255.65	263.56
349.81	330.53	251.14	259.92	225.07	240.37	228.28	233.46	233.09
288.88	247.05	249.5	204.18	196.48	219.02	207.35	215.42	220.73
178.14	176.83	214.53	167.94	158.76	170.1	161.02	170.07	148.09
165.01	142.9	208.51	160.92	145.97	148.54	149.27	146.11	147.16
137.27	131.98	162.5	152.92	138.85	142.1	143.52	140.31	141.4
120.59	113.23	146.86	135.55	123.16	117.52	114.39	122.47	112.27
103.66	92.36	107.97	116.18	101.77	106.88	107.27	111.42	97.27
75.18	70.83	92.49	103.31	98.23	91.21	95.26	89.93	92.87
60.66	62.23	85.54	87.03	79.11	84.44	74.67	78.94	83.81
39.12	37.24	73.55	75.45	64.75	73.79	60.17	74.44	68.84
18.78	32.85	66.78	46.96	45.07	34.95	16.03	37.23	41.67
26.76i	23.71i	38.84	41.29	24.25i	20.5	7.86i	22.25	23.31
54.07i	64.79i	9.83	14.15	55.57i	13.38	16.84i	15.15i	22.33i
0_2	1_3	2_3	5_3	2_3	1_4	4_3	3_2	3_5
d	d	d	d	f	d	d	d	d
3781.87	3773.24	3749.67	3743.34	3742.17	3730.72	3782.88	3804.87	3739.4
3100.41	3101.11	3086.99	3094.64	3093.01	3104.93	3103.59	3092.82	3094.54
3071.63	3073.69	3060.83	3069.17	3071.46	3036.03	3071.83	3075.11	3071.12
3064.89	3065.02	3042.03	3038.43	3052.13	3034.34	3066.6	3070.69	3050.1
2959.64	2959.6	2946.86	2945.19	2949.32	2943.96	2959.71	2963.29	2949.12
1526.64	1519.76	1479.89	1489.69	1500.94	1484.35	1516.12	1526.49	1501.83
1517.01	1508.93	1464.64	1462.07	1497.39	1409.28	1513.21	1520.03	1489.24
1310.48	1305.65	1271.25	1277.65	1281.98	1279.59	1314.88	1292.93	1283.84
1296.54	1288.98	1262.6	1265.82	1278.1	1264.67	1295.03	1284.59	1272.61
1267.88	1267.08	1256.72	1260.41	1266.27	1224.77	1261.36	1281.35	1271.15
471.55	466.76	465.04	464.85	467.7	465.29	473.34	474.06	470.95
388.32	399.78	385.45	385.24	399.17	415.98	395.36	369.2	410.25
346.31	339.96	333.51	323.03	331.17	382.96	359.55	274.4	336.17
258.15	241.05	241.4	244.85	247.49	253.75	255.22	255.11	256.97

Continued on next page

235.91	231.29	217.39	214.93	218.57	237.5	240.4	247.24	226.69
227.5	221.87	171.01	184.9	184.72	219.32	230.62	173.59	197.0
148.74	143.6	151.62	167.69	171.44	150.04	147.55	151.46	171.34
145.16	141.02	140.05	142.39	141.12	140.69	144.74	148.81	144.45
137.28	139.08	138.26	139.8	139.15	138.08	136.77	136.04	141.13
115.25	113.55	114.8	128.23	124.92	116.72	108.55	119.66	116.27
102.05	97.51	106.12	117.15	115.62	104.63	94.03	105.97	112.17
87.78	81.2	72.14	95.2	70.15	79.6	87.49	74.65	79.32
82.19	78.61	62.4	78.61	60.37	74.43	79.22	68.9	70.49
67.0	61.62	41.69	64.23	44.28	51.75	57.05	50.78	48.07
30.7	43.28	36.11	56.43	33.04	24.66	32.01	42.89	38.72
13.01	16.02	19.97	26.86	10.25i	19.7	22.48	11.46	23.0
21.27i	27.99i	16.88i	13.07i	35.93i	31.87i	5.0i	17.89i	12.3i
3_4	4_5	0_3	3_0	1_0	1_2	1_2	5_2	
d	u	d	d	d	u	f	u	
3753.95	3796.08	3765.82	3741.11	3736.96	3718.06	3750.34	3746.41	
3082.25	3102.67	3102.71	3085.87	3097.63	3101.65	3097.21	3101.25	
3063.61	3074.62	3072.19	3063.02	3073.02	3070.29	3071.23	3072.7	
3044.73	3067.89	3066.78	3044.95	3048.39	3059.08	3061.83	3061.05	
2943.41	2963.02	2958.94	2944.8	2950.27	2955.85	2957.7	2957.55	
1483.49	1523.42	1520.53	1490.41	1501.85	1524.65	1526.42	1525.82	
1459.26	1515.93	1513.91	1449.15	1480.08	1520.21	1522.09	1522.05	
1274.03	1310.97	1312.32	1282.79	1277.73	1304.38	1297.43	1302.49	
1268.29	1295.71	1292.04	1260.97	1275.11	1295.12	1292.72	1294.68	
1253.26	1266.22	1265.15	1244.43	1267.13	1266.63	1272.74	1271.42	
466.77	468.69	474.78	464.99	468.58	471.75	471.32	474.05	
355.45	378.24	406.27	364.63	395.52	428.12	401.89	428.28	
335.32	338.15	360.3	354.95	340.01	344.55	318.6	338.44	
243.47	257.16	248.74	245.05	251.2	308.89	303.15	289.35	
222.1	234.86	241.6	217.8	223.05	233.26	206.47	238.32	
163.17	217.29	230.79	166.86	198.54	206.11	203.52	227.79	
148.28	146.86	150.75	142.36	173.23	166.34	161.8	194.34	
141.54	140.52	143.66	140.33	140.25	164.14	148.62	171.37	
140.37	131.16	132.36	139.7	138.42	149.91	143.91	143.07	
109.64	108.82	113.14	135.55	118.76	139.94	134.06	134.0	
94.86	97.4	88.02	121.77	106.03	110.02	107.59	118.48	
78.86	83.23	83.31	76.66	75.1	104.8	97.33	111.6	
58.38	75.09	71.65	65.43	46.49	83.6	69.54	77.6	
45.2	49.33	48.97	40.6	30.37	58.52	54.99	69.75	
36.03	26.29	37.9	22.77	16.48i	43.38	27.97	50.7	
17.79i	20.3	18.7	27.29i	23.54i	22.09	3.95i	44.06	
32.18i	21.31i	31.56i	39.49i	46.68i	32.73i	26.25i	23.81	

3 Stability of the $NiF_2(F_2)$ (001) surface

Figure S6 shows the surface energy of different surface cuts of NiF_2 versus the potential calculated via the computational hydrogen electrode. The surface model under investigation in this work is denoted by orange dashed line. A detailed explanation of how the surface energies are calculated and detailed discussion of other surface cuts can be found in reference [22]. Potentials at which the Ni(III) and Ni(IV) surfaces are stabilised are marked with arrows.

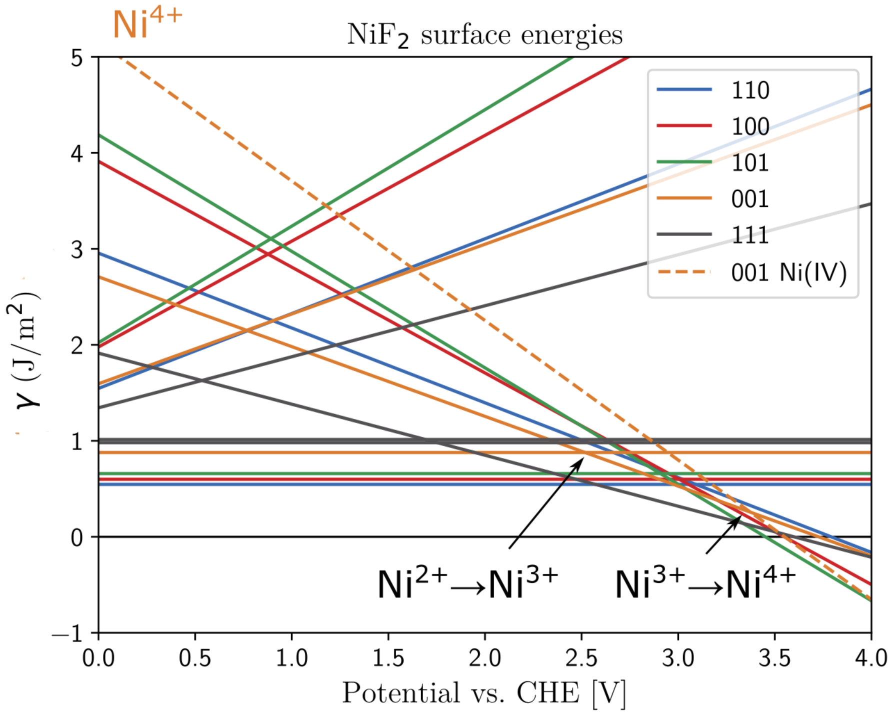

Figure S6: Surface energies of different cuts of NiF_2 surface versus the potential calculated via the computational hydrogen electrode. The surface energies of the Ni^{2+} to Ni^{3+} transition are taken from reference [22]. The same methodology as in this reference was applied to calculate the surface energy of $NiF_2(F_2)$ (001) surface, shown with dashed line.

4 Plot of imaginary vibrational mode from NEB

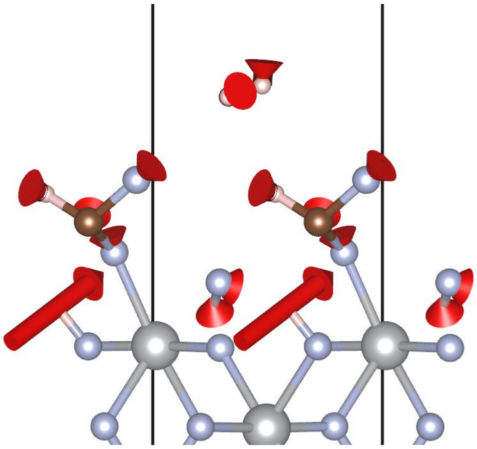

Figure S7: Imaginary vibrational mode displacements of the calculated transition state (image 4 in Figure 7 in the manuscript), with movement of atoms depicted by red arrows.
